# Cephalopods in neuroscience: regulations, research and the 3Rs

**DOI:** 10.1007/s10158-013-0165-x

**Published:** 2014-01-03

**Authors:** Graziano Fiorito, Andrea Affuso, David B. Anderson, Jennifer Basil, Laure Bonnaud, Giovanni Botta, Alison Cole, Livia D’Angelo, Paolo De Girolamo, Ngaire Dennison, Ludovic Dickel, Anna Di Cosmo, Carlo Di Cristo, Camino Gestal, Rute Fonseca, Frank Grasso, Tore Kristiansen, Michael Kuba, Fulvio Maffucci, Arianna Manciocco, Felix Christopher Mark, Daniela Melillo, Daniel Osorio, Anna Palumbo, Kerry Perkins, Giovanna Ponte, Marcello Raspa, Nadav Shashar, Jane Smith, David Smith, António Sykes, Roger Villanueva, Nathan Tublitz, Letizia Zullo, Paul Andrews

**Affiliations:** 1 Stazione Zoologica Anton Dohrn, Villa Comunale, Naples, Italy; 2 Associazione Cephalopod Research ‘CephRes’, ONLUS, Via dei Fiorentini 21, 80133 Naples, Italy; 3 Animal Model Facility, BIOGEM SCARL, Via Camporeale Area PIP, Ariano Irpino, AV Italy; 4 Midlothian Innovation Centre, Pentland Management Systems, Pentlandfield, Roslin, EH25 9RE UK; 5 Biology Department, CUNY Graduate Center, Brooklyn College, 2900 Bedford Avenue, Brooklyn, NY 11210 USA; 6 Muséum National d’Histoire Naturelle, DMPA, Lab. BOREA, UMR MNHN CNRS 7208-IRD 207-UPMC, Paris Cedex, France; 7 Université Paris Diderot, Sorbonne Paris Cité, Paris, France; 8 Ministero della Salute, Via G. Ribotta 5, 00144 Rome, Italy; 9 BIOGEM SCARL, Via Camporeale Area PIP, Ariano Irpino, AV Italy; 10 Department of Veterinary Medicine and Animal Productions, University of Naples Federico II, Naples, Italy; 11 Home Office, Animals in Science Regulation Unit, Dundee, DD1 9WW Scotland, UK; 12 Groupe Mémoire et Plasticité Comportementale, EA4259, GdR CNRS 2822 Ethology, University of Caen Basse-Normandy, Caen, France; 13 Department of Biology, University of Naples Federico II, Naples, Italy; 14 Department of Biological and Environmental Sciences, University of Sannio, Benevento, Italy; 15 Instituto de Investigaciones Marinas (IIM-CSIC), Vigo, Spain; 16 Center for GeoGenetics, University of Copenhagen, 1350 Copenhagen, Denmark; 17 Department of Psychology, BioMimetic and Cognitive Robotics, Brooklyn College, CUNY, Brooklyn, NY 11210 USA; 18 Institute of Marine Research, 5817 Bergen, Norway; 19 Max Planck Institute for Brain Research, 60438 Frankfurt, Germany; 20 Institute of Cognitive Sciences and Technologies, CNR, Via Aldovrandi 16b, Rome, Italy; 21 Integrative Ecophysiology, Alfred Wegener Institute for Polar and Marine Research, Bremerhaven, Germany; 22 School of Life Sciences, University of Sussex, Brighton, E Sussex, BN1 9RH UK; 23 European Mouse Mutant Archive (CNR-EMMA), Consiglio Nazionale delle Ricerche, Campus A. Buzzati-Traverso, Viale E. Ramarini, 32, 00015 Monterotondo Scalo, Roma, Italy; 24 Department of Life Sciences, Ben-Gurion University of the Negev, Eilat Campus, 84105 Beer-Sheva, Israel; 25 Federation for Laboratory Animal Science Associations (FELASA), London, UK; 26 The Boyd Group, Hereford, UK; 27 Centre of Marine Sciences (CCMAR), Universidade do Algarve, 8005-139 Faro, Portugal; 28 Renewable Marine Resources Department, Institut de Ciències del Mar (CSIC), Barcelona, Spain; 29 Department of Biology, University of Oregon, Eugene, OR 97403 USA; 30 Department of Neuroscience and Brain Technologies, Istituto Italiano di Tecnologia, Genoa, Italy; 31 Division of Biomedical Sciences, St George’s University of London, Cranmer Terrace, London, SW17 0RE UK

**Keywords:** Cephalopods, Directive2010/63/EU, Animal welfare, 3Rs, Neuroscience

## Abstract

Cephalopods have been utilised in neuroscience research for more than 100 years particularly because of their phenotypic plasticity, complex and centralised nervous system, tractability for studies of learning and cellular mechanisms of memory (e.g. long-term potentiation) and anatomical features facilitating physiological studies (e.g. squid giant axon and synapse). On 1 January 2013, research using any of the about 700 extant species of “live cephalopods” became regulated within the European Union by Directive 2010/63/EU on the “Protection of Animals used for Scientific Purposes”, giving cephalopods the same EU legal protection as previously afforded only to vertebrates. The Directive has a number of implications, particularly for neuroscience research. These include: (1) projects will need justification, authorisation from local competent authorities, and be subject to review including a harm-benefit assessment and adherence to the 3Rs principles (Replacement, Refinement and Reduction). (2) To support project evaluation and compliance with the new EU law, guidelines specific to cephalopods will need to be developed, covering capture, transport, handling, housing, care, maintenance, health monitoring, humane anaesthesia, analgesia and euthanasia. (3) Objective criteria need to be developed to identify signs of pain, suffering, distress and lasting harm particularly in the context of their induction by an experimental procedure. Despite diversity of views existing on some of these topics, this paper reviews the above topics and describes the approaches being taken by the cephalopod research community (represented by the authorship) to produce “guidelines” and the potential contribution of neuroscience research to cephalopod welfare.

## Introduction

Cephalopods are a numerically small but significant taxon of invertebrates (phylum Mollusca) whose richness of behavioural capabilities (Borrelli and Fiorito 2008) fascinate the public and researchers alike, but that also represent a very important resource for human consumption (Jereb et al. 2005). The class Cephalopoda is considered the most complex one in the phylum Mollusca and arguably amongst all other invertebrate phyla as reflected in the use of the term “advanced invertebrate” or “exceptional invertebrate class” (sensu Zullo and Hochner 2011). It includes exclusively marine living species considered to have rivaled fishes during evolution (e.g. Packard 1972; but see also Kröger et al. 2011). Cephalopods demonstrate a refined and extraordinary ability to adapt their morphology (Kröger et al. 2011) and behavioural repertoire to their niche; this may have contributed greatly to their success (Hochner et al. 2006; Hochner 2008, 2012; Borrelli and Fiorito 2008). Amongst the several notable expressions of phenotypic plasticity in cephalopods (Hanlon and Messenger 1996; see also Barbato et al. 2007) is the capability to display environmentally cued phenotypes, i.e. body patterns (Borrelli et al. 2006). The complex behavioural and learning capabilities of cephalopods (Hanlon and Messenger 1996; Borrelli and Fiorito 2008; Huffard 2013) correspond to a highly sophisticated nervous system that appears to be correlated with their lifestyle (Nixon and Young 2003; Borrelli 2007). The flexibility of the behavioural repertoire of cephalopods is supported by evident cellular and synaptic plasticity at the level of the central and peripheral nervous system and of the neuromuscular junctions (review in Brown and Piscopo 2013). Cephalopods are well known amongst neuroscientists for their contribution to fundamental understanding of the nervous system (Young 1985; Abbott et al. 1995; but see also Brown and Piscopo 2013).

This paper is prompted by the recent inclusion of “all live cephalopods” in Directive 2010/63/EU that regulates the use of animals for scientific purposes (European Parliament and Council of the European Union 2010).

### Regulation of scientific uses of cephalopods

National legislation regulating experimentation on living animals began to appear in several European countries in the late nineteenth century and made a division between vertebrates and invertebrates, with only vertebrates being regulated (i.e.: United Kingdom, 1876; Germany, 1883; Denmark, 1891; see Smith et al. 2013 for references).^1^

One species of cephalopod, *Octopus vulgaris*, was included in a revision of the UK legislation (Animals [Scientific Procedures] Act 1986), but no studies have ever been conducted under the legislation. Cephalopods have been included in various national codes of practice and legislation covering research in several countries outside the EU, for example: Canada, 1991; New Zealand, 1999; Australia, 2004; Switzerland, 2011; Norway, 2011; see Smith et al. (2013) for details and references.

Animal experimentation involving all vertebrates has been regulated at EU level since 1986 (Directive 86/609/EEC) and Directive 2010/63/EU (European Parliament and Council of the European Union 2010), which we will refer to here as the “Directive” is a major revision intended to make the regulation “more stringent and transparent” as well as recognising advances in research techniques, improved understanding and assessment of animal welfare (see: Broom 1991a, b, 2011 for an introduction to the issues) and developments in ethical review of animal experimentation (Smith et al. 2013) particularly in relation to invertebrates (Mather and Anderson 2007; Moltschaniwskyj et al. 2007; Horvath et al. 2013). The Directive also places particular emphasis on application of the “3Rs” principles of Replacement, Reduction and Refinement formulated by Russell and Burch (1959) and discussed in detail below in relation to neuroscience research.

For invertebrate research in the EU, Directive 2010/63/EU which implemented on 1 January 2013 marks a paradigm shift by covering the use of an entire class of Molluscs, namely “live cephalopods” (i.e. hatched juveniles and adults) in the legislation covering experimental procedures likely to cause pain, suffering, distress or lasting harm (EFSA Panel on Animal Health and Welfare 2005; European Parliament and Council of the European Union 2010; Smith et al. 2013). This means that, under the Directive and transposed national laws, cephalopods have the same legal status as vertebrates in relation to their experimental use in research and testing (Smith et al. 2013).

It should be noted that drafts of the Directive also included decapod Crustacea (e.g. crabs, lobsters). Although decapod crustaceans were not included in the adopted Directive, it is likely that this issue will be revisited because of the continuing debate about their pain perception (Gherardi 2009; Magee and Elwood 2013; Horvath et al. 2013) and also because as was the case with cephalopods there is interest in this issue from animal welfare and animal rights groups (Advocates for Animals 2005).

The decision to include cephalopods was based primarily upon the recommendations of a scientific panel which concluded that there was “scientific evidence of their ability to experience pain, suffering, distress and lasting harm” (i.e. PSLDH; Directive 2010/63/EU: Recital 8, European Parliament and Council of the European Union 2010). However, note that this view is not universally shared by the global research community. In essence, much of the evidence for inclusion of cephalopods in the Directive is based upon various aspects of neuroscience research on cephalopods and the criteria used, as well as additional recent studies, are reviewed by Andrews et al. (2013).

It is anticipated that the Directive will provide a stimulus to cephalopod neuroscience research, as ensuring the highest welfare standards requires answers to a number of questions some of which are summarised in Table 1.

**Table 1:** Possible areas of biological and neuroscience research expected to contribute to increasing knowledge of cephalopod welfare as stimulated by Directive 2010/63/EU

***Optimal conditions of care and maintenance of animals also aimed to increase well-being***
***Evidence of the capacity for cephalopods to experience pain***
Search for receptors sensitive to noxious stimuli
Functional analysis of “brain centres”
Analysis of nervous pathways connecting the nociceptive system to higher “brain centres”
Search for receptors for opioid, cannabinoid and analgesic steroid substances
Studies on analgesia and animals’ responses
Behavioural and functional analysis of animals’ response to painful stimuli
Search of objective signs of pain, suffering and distress
Physiological indicators of pain
***Humane end points in cephalopod studies***
***General anaesthesia for cephalopods***
Establishment of objective criteria for assessing depth of general anaesthesia
Methods for maintenance of general anaesthesia and facilitation of recovery
Methods for production of local anaesthesia and systemic analgesia
***Methods for humane killing***
***Physiological analysis and evaluation of stress, suffering or pain, including evaluation of biomarkers of immune response linked to diseases and distress***
***Noninvasive approaches to characterise physiological function of organs and systems and monitoring effects of experimental treatments***

For review and further discussion see Andrews (2011a, b), Andrews et al. (2013) and Smith et al. (2013). See also: Borrelli and Fiorito (2008), Ponte and Fiorito (2011, 2013), Boal (2011), Margheri et al. (2011b), Ponte et al. (2013)

The Directive will impact upon scientific work using any of the approximately 700 extant species of cephalopods, but in practice within the EU the species most commonly used are the coleoid cephalopods: the cuttlefish *Sepia officinalis*; the squids *Loligo vulgaris* and *Loligo forbesi*; and the octopuses *O. vulgaris*, *Eledone cirrhosa and Eledone moschata*. The shelled cephalopod *Nautilus pompilius* is also used occasionally but is imported from tropical waters.

Compliance with the new EU legislation will be challenging for many areas of cephalopod research, especially neuroscience; some concern has already been expressed regarding the applicability of “mammal-centric” regulations to cephalopods (Nosengo 2011). Yet, the legislation by itself is not aimed to be “mammal-centric”, as the law applies equally to fish, amphibians and birds as well as mammals, and the principles are the same for all species!

## Some implications of the Directive for research on cephalopods

The inclusion of cephalopods in the Directive has a number of implications for different groups:

1. *Researchers* All researchers who use cephalopods in their research will need to ascertain whether the intended experiments are covered by the Directive and if so an application will need to be submitted to the appropriate National Competent Authority (NCA;^2^ e.g. Home Office in the UK; Ministère de l’Enseignement Supérieur et de la Recherche in France; Ministero della Salute in Italy) and approval obtained prior to starting the project. The authorisation process involves impartial evaluation of the project by the NCA including examination of the purpose of the research procedures (permitted purposes are listed in Article 5 of the Directive), compliance with 3Rs, severity classification of procedures and a harm-benefit analysis of the project (Voipio et al. 2004; for details and examples see: Smith et al. 2013). Researchers should consult their NCA to obtain details of the authorisation process as although the principles are common throughout the EU, the way in which the Directive is transposed into national legislation may differ. It should also be noted that in addition to covering the experiments themselves, the Directive also regulates the place where experiments are undertaken, the standards of housing and care of animals used for research and methods of euthanasia. Researchers will also need to ensure that their project is authorised and that their whole team is familiar with the national law covering their experiments are appropriately trained and competent to perform the procedures (Article 23 of the Directive) and, if required by the national legislation, that the project and personnel are covered by appropriate licences (e.g. in the UK Home Office Project and Personal Licences). A checklist of what is needed in the case of conducting cephalopod research in the EU is summarised in Smith et al. (2013).
2. *Animal technologists, veterinarians and regulators* The Directive places the care and welfare within a legal framework requiring documented monitoring and compliance. Research on cephalopods, under the Directive, is likely to be performed in the same places where research is currently undertaken, so those currently responsible for care and welfare will be hopefully familiar with the expected requirements. Nonetheless, it is likely that some training will be needed even for those familiar with maintenance of cephalopods in the laboratory. In addition, veterinarians or other suitable qualified experts with responsibility for laboratory animal facilities will need to become familiar with all aspects of health and welfare of the cephalopod species in their care. Although there are reviews covering cephalopod health (e.g.: Boletzky and Hanlon 1983; Hochberg 1990; Hanlon and Forsythe 1990a, b; Boyle 1991; Castellanos-Martinez and Gestal 2013), there are few aquatic medicine courses covering invertebrates (see for example: Virginia-Maryland Regional College of Veterinary Medicine, http://www.vetmed.vt.edu/research/aquatic/education.html).
  One aspect of monitoring compliance with the Directive involves “regular inspections” of establishments, of which “an appropriate proportion” is to be carried out “without prior warning” (Directive Article 34). Monitoring may involve inspection of the place where the animals are kept, observations of procedures and inspection of experimental records. The records must include the source of the animals, whether they were purpose bred, what they were used for and by whom, and their fate at the end of the study (Directive Article 30). Those responsible for monitoring compliance with the Directive will need training to become familiar with this newly regulated class of animal.
3. *Funders* Most grant funding agencies and charities already require that grant applications involving research on vertebrates certify that, if required, appropriate authorisation (normally including “ethical” review) to conduct the proposed studies is in place. As cephalopods are now covered by the same legislation as vertebrates, grants involving particular types of research concerning their regulated use will need to ensure that the proposed studies comply with the Directive and any national Codes of Practice related to care and welfare.
4. *Journal editors and reviewers* The editors and reviewers of Journals will need to be made aware of the change in the regulation within the EU to ensure that papers submitted for publication if appropriate make reference to compliance with the Directive. This may be difficult for a short period as although the Directive was implemented on 1 January 2013, some EU states have not yet transposed it into national legislation (http://ec.europa.eu/environment/chemicals/lab_animals/transposition_en.htm). Although not part of the Directive, several journals (e.g. Nature, PLoS)^3^ have voluntary adopted the ARRIVE (Animal Research: Reporting of in vivo Experiments: http://www.nc3rs.org.uk/page.asp?id=1357) guidelines for reporting experiment (Kilkenny et al. 2010). These guidelines provide checklists of information that should be included in published papers, particularly in the methods sections. Whilst many papers involving cephalopods already contain much of this information, key information is lacking in others. For example, only in the 40 % of papers published in the 2010 (*n* = 65; source WoK: ISI Web of Knowledge), mention the conditions in which cephalopods are maintained. However, only half of those (13 out 26 papers) provide details on tank and lighting. Further analysis reveals that for the five cases in which octopuses were utilised, tanks ranged from 200 to 7,000 L and for cuttlefishes (*n* = 7) a wider range of tank sizes was utilised (from 30 to 20,000 L). It is remarkable that a justification for such a diversity of approach for accommodating animals is missing in the papers. Finally, no indication of the stocking density of animals is provided in the great majority of studies here considered.
  The lack of such information makes it difficult to undertake systematic analysis of housing conditions in order to derive guidelines reflecting the consensus in the literature. In addition, lack of critical information on sex, body weight, feeding, tank size, lighting, handling and euthanasia methodology can compromise assessment of results. Based upon studies in vertebrates, the outcome of neuroscience studies, and in particular studies of behaviour in cephalopods, is most likely to be sensitive to environmental factors.
5. *The public* Although cephalopods are frequently portrayed as creatures of nightmares in films and literature (e.g.: Muntz 1995; Ellis 1998), people are nevertheless fascinated by these animals in display aquaria and they make frequent appearances in natural history documentaries and the media. In contrast to mice, rats and rabbits, the public do not make an immediate association between cephalopods and “animal experimentation”, but this may change as the knowledge of their inclusion in the Directive becomes more widely known and researchers should be aware that their studies may come under public and media scrutiny.

## Neuroscience research and the impact of the Directive

Cephalopods are a large group of marine predators whose major aspects of biology, behaviour, and ecology provide a backdrop against which their neurobiology can be interpreted. Special features of their reproduction (Rocha et al. 2001), camouflage, motor control, memory, learning, and behavioural ecology may be considered as special cases of convergent evolution with vertebrates (Packard 1972; Borrelli and Fiorito 2008; Huffard 2013). Neuroscience research involving brain and behaviour is particularly prominent because of the perceived status of cephalopods as “advanced invertebrates”. Cephalopods are model organisms for a diverse range of neuroscience areas, and their anatomical features provide unique opportunities for research (see examples in Table 2). Neuroscience research studies may be particularly impacted by the Directive as they cover a diversity of experimental techniques (“procedures”) which are often invasive and may cause pain, suffering, distress and lasting harm. This aspect is discussed in detail hereunder with examples of the types of study likely to fall within the scope of the Directive and which will need to be authorised by the appropriate national competent authority. Although researchers should be familiar with all the requirements of the Directive in relation to routine care and welfare, it is the aspects of the Directive covering procedures and their impact upon the health and welfare of the animal that are likely to have the greatest impact upon their use in research.

**Table 2:** A selected summary of cephalopod neuroscience and neurobehavioural research [for review see also: Borrelli and Fiorito (2008), Brown and Piscopo (2013), Huffard (2013)]

**Squid giant axon and giant synapse**
Physiology of resting membrane potential and action potential [consider also the Nobel Prize to Eccles (Hodgkin and Huxley^1952^)]
Giant axon-Schwann cell signalling
Physiology and pharmacology of synaptic transmission
Axoplasmic transport
Consider also recent studies on
The effect of mutant SOD1 implicated in Lou Gehrig disease in humans
Effect of human tau-protein implicated in Alzheimer’s disease
Relevant references: Young (1938), Bullock (1948), Hodgkin and Huxley (1952), Bloedel et al. (1966), Coles and Abbott (1996), Moreno et al. (2011), Song et al. (2012)
**Behavioural studies and the search for their neural correlates**
Behavioural plasticity, learning and memory
Sleep-like states
Consciousness
Physiology and pharmacology of long-term potentiation (LTP)
Relevant references: Sanders (1975), Fiorito et al. (1990), Young (1991, 1995), Fiorito and Scotto (1992), Robertson et al. (1994, 1995, 1996), Fiorito and Chichery (1995), Boal 1996, Boal and Gonzalez (1998), Boal and Golden (1999), Boal et al. (2000), Agin et al. (2001), Vinogradova et al. (2002), Agin et al. (2003), Hochner et al. (2003, 2006), Karson et al. (2003), Darmaillacq et al. (2004, 2006), Boal (2006), Agin et al. (2006), Brown et al. (2006), Langridge et al. (2007), Hochner (2008), Shomrat et al. (2008, 2010), Mather (2008), Edelman and Seth (2009), Zullo et al. (2009), Zylinski et al. (2011), Shomrat et al. (2011), Tricarico et al. (2011), Zullo and Hochner (2011), Edelman (2011), Osorio and Zylinski (2011), Gutnick et al. (2011a, b), Josef et al. (2012), Hochner (2012), Frank et al. (2012)
**Neurotransmitters** (sensu lato)
Relevant references: Florey (1963), Loe and Florey (1966), Florey and Winesdorfer (1968), Tansey (1978, 1979), Budelmann and Bonn (1982), Williamson (1989), Cornwell et al. (1993), Messenger (1996), Palumbo et al. (1999), Loi and Tublitz (2000), Lima et al. (2003), Di Cosmo et al. (2004, 2006, 2007), Fiore et al. (2004), Scheinker et al. (2005), Di Cristo et al. (2007), Boyer et al. (2007), Wollesen et al. (2008, 2010a, b, 2012), Bardou et al. (2009, 2010), Shomrat et al. (2010), Ponte (2012), Conti et al. (2013)
**Nociception**
Relevant references: Crook and Walters (2011), Crook et al. (2011, 2013), Hague et al. (2013), Andrews et al. (2013), but see also: Wells et al. (1965), Wells (1978), Hanlon and Messenger (1996), Mather and Anderson (2007)
**Regeneration**
Regeneration of appendages following damage (wild and experimental)
Nerve regrowth
Relevant references: Lange (1920), Sereni and Young (1932), Sanders and Young (1974), Féral (1988), Rohrbach and Schmidtberg (2006), Florini et al. (2011), Fossati et al. (2013)
**Neuromotor control**
Motor and sensory control of arm movements
Arm use preference and functioning (including suckers)
Octopus arm as a bio-inspired robotic model
Control of chromatophores and body patterning
Relevant references: Kier (1982, 1985, 1991), Kier and Smith (1985), Hanlon and Messenger (1996), Kier and VanLeeuwen (1997), Mather (1998), Loi and Tublitz (2000), Messenger (2001), Sumbre et al. (2001, 2005, 2006), Borrelli et al. (2006), Gutfreund et al. (2006), Byrne et al. (2006a, b), Grasso and Setlur (2007), Barbato et al. (2007), Grasso (2008), Kier and Schachat (2008), Zullo et al. (2009), Mattiello et al. (2010), Calisti et al. (2011), Margheri et al. (2011a, b, 2012), Mazzolai et al. (2012), Laschi et al. (2012)
**Physiology of the sensory systems**
Visual and chemo-tactile systems
Statocyst and oculomotor systems
Relevant references: Bullock (1965), Williamson (1986, 1989, 1995), Budelmann (1995), Abbott et al. (1995), Lucero and Gilly (1995), Budelmann et al. (1997), Williamson and Chrachri (2004)
**Development and functional organisation of the “brain” and muscles**
Relevant references: Young (1991, 1995), Gutfreund et al. (1996), Shigeno et al. (2001a, b, 2008a, b), Callaerts et al. (2002), Shigeno and Yamamoto (2002), Lee et al. (2003), Hartmann et al. (2003), Nixon and Young (2003), Grimaldi et al. (2004), Borrelli (2007), Navet et al. (2008), Lee et al. (2009), Baratte and Bonnaud (2009), Navet et al. (2009), Wollesen et al. (2009), Zullo et al. (2009), Zullo and Hochner (2011), Hochner (2012), Mattiello et al. (2012)

An annotated bibliography on classical contributions to cephalopod’ biology and physiology is also provided by Ponte et al. (2013). References to relevant studies included are given as examples

## Care and welfare of cephalopods in neuroscience research and the need for guidelines

The inclusion of all live cephalopods (i.e. larval and adult forms) in the new EU Directive has a number of practical implications for those undertaking research involving cephalopods, irrespective of the subject area. Guidelines for the general care and welfare for vertebrate laboratory species such as mammals (Sikes and Gannon 2011) and fishes (DeTolla et al. 1995; Hawkins et al. 2011a) are well developed, and specific guidelines are available for the welfare of vertebrates used in particular types of research such as cancer (Workman et al. 2010). For vertebrates in general and mammals specifically, objective criteria for identification and assessment of pain, suffering, distress and lasting harm are well researched (e.g.: Morton and Griffiths 1985; Bateson 1991) and protocols for surgery, anaesthesia, analgesia and humane euthanasia established. However, for cephalopods such knowledge is relatively rudimentary and maybe further hampered by lack of specific veterinary expertise; as in contrast to vertebrates, cephalopods are not common companion animals, although they are often found in display aquaria and knowledge gained in this setting is making a useful contribution to understanding their general welfare requirements.

The Directive is likely to stimulate research in the above areas so as to facilitate development of evidence-based guidelines for optimal care and welfare (Moltschaniwskyj et al. 2007; Louhimies 2011; see for example: Andrews 2011a, b; Goncalves et al. 2012; Sykes et al. 2012; Andrews et al. 2013; Smith et al. 2013).

The text of the Directive does not provide specific guidance on the above aspects for cephalopods, and at present, there are no national codes of practice for care and use of cephalopods under the terms of the Directive. In view of this, the cephalopod research community initiated a project to develop guidelines for the Care and Welfare of Cephalopods in Research. This project is an initiative^4^ between the Federation of European Laboratory Animal Science Association (FELASA: www.felasa.eu), the Boyd Group (http://www.boyd-group.demon.co.uk/) and CephRes (www.cephalopodresearch.org). The guidelines are being developed based upon structured discussions amongst 30 active cephalopod researchers drawn from 26 research institutes in 11 countries including from outside the EU. The discussions also included national and EU legislators and regulators, as well as researchers with expertise in vertebrate animal welfare (i.e. Giovanni Botta, Italy; Paolo De Girolamo, Italy; Ngaire Dennison, UK; Tore Kristiansen, Norway; Marcello Raspa, Italy; Jane Smith, UK; David Smith, UK). Some of the main points arising from these discussions, with particular impact upon neuroscience research, are discussed below. It must be emphasised that these only provide an overview, and there are still many areas of contention. More detailed reviews of specific aspects and species should be consulted for more practical information.

Table 3 summarises some of the main reviews and topics in this area. However, given that there are more than 700 known living species of cephalopods of which a wide variety are used for scientific purposes, care should be taken to meet the particular requirements of individual species involved in experiments or other scientific procedures. Species-specific guidelines will need to be developed, and for many aspects of care and welfare, this will require research, but here we focus on the more generic issues relating to the cephalopod species most commonly used in the EU, as a baseline for future Guideline development.

**Table 3:** Summary of resources relevant to implementation and compliance with specific aspects of Directive 2010/63/EU in relation to cephalopods

Area covered by the Directive	References
Biology including normal behaviour and physiology	Bullock (1965), Wells (1962, 1978), Hanlon and Messenger (1996), Norman (2000), Boyle and Rodhouse (2005), Borrelli et al. (2006), Boal (2011)
Overview of Directive requirements and project (“ethical”) review	Smith et al. (2013)
List of what needs to be done if you are a researcher	
Ethics of cephalopod research and invertebrates in general	Mather and Anderson (2007), Moltschaniwskyj et al. (2007), Andrews (2011a), Horvath et al. (2013)
3Rs principles in relation to cephalopod research including worked examples of project review	Smith et al. (2013)
Various aspects of general maintenance, handling, rearing and culture of a number of cephalopod species	Grimpe (1928), Walker et al. (1970), Boletzky and Hanlon (1983), Boal (2011), Sykes et al. (2012)
Pain, suffering and distress in cephalopods	Crook and Walters (2011), Crook et al. (2011, 2013), Andrews et al. (2013)
Approaches to objective measurement of cephalopod health and welfare	
General anaesthesia	Gunkel and Lewbart (2008), Pagano et al. (2011), Lewbart and Mosley (2012), Goncalves et al. (2012), Gleadall (2013), Andrews et al. (2013)
Euthanasia	Boyle (1991), Demers et al. (2006), Andrews et al. (2013)

## Care and welfare of cephalopods: an introduction

This section discusses some of the key areas covered by the Directive and which we believe impact particularly upon neuroscience research involving cephalopods.

### Sources of animals

Cephalopods used in research are currently commonly taken from the wild (for review on fishing methods see: Lane 1960; Boyle and Rodhouse 2005) mainly because of the difficulties of laboratory breeding of many but not all species. Recent exceptions are, for example, *S. officinalis*, *Octopus bimaculoides*, *Euprymna scolopes* (review in Albertin et al. 2012). However, the Directive (Article 9) prohibits capture in the wild *unless* an exemption has been granted by the NCA. In practice, this means that animals may still be obtained from the wild provided that this can be justified to the Competent Authority. In addition, capture must be undertaken by competent persons using methods which do not cause pain, suffering, distress or lasting harm.

Wild caught animals may be obtained from approved suppliers (including authorised laboratories specialising in cephalopod research or specialist importers as in the case of *Nautilus*), but they must also obtain approval for the capture from the NCA.

Depending upon the research project, one potential issue with using research animals from the wild is that it may be harder to ensure “standardised” groups of animals both within a study continuing over several years and to permit comparison between research groups in different locations. This inherent variability may lead to the use of a larger number of animals than in other studies to demonstrate statistically significant effects, particularly in behavioural studies, and this could become an issue in project evaluation and authorisation where factors taken into account include animal numbers (estimates may include power calculation) and experimental design (including statistical analysis) to ensure that the minimum number of animals are used to achieve the scientific objective (see instructions and citations included in Animal Behaviour 2012).

### Transport, quarantine and acclimatisation

Transport of animals should be minimised, and where possible the researcher should travel to study the animals not vice versa. A solution is to transport eggs rather than animals (e.g. cuttlefish) and to culture these; however, as mentioned above, this is not possible for most cephalopod species. Transport of animals should always be in sea water. The levels of available oxygen and accumulation of metabolites in a limited volume are important considerations for transport of living cephalopods, as recommended in the classic work by Grimpe (1928).

When animals are transported, the potential impact upon their health and welfare will need to be assessed and careful consideration given to the time required for adaptation before experimentation. On arrival in the laboratory, all animals should be closely inspected for overt signs of illness and if necessary advice sought from the person with legal responsibility for the care of animals (e.g. veterinarian or other appropriately qualified expert) on action to be taken.

Quarantining the animals is good practice whether they come from the wild or an authorised breeder/supplier as it reduces the risk of introducing infectious agents or parasites that could spread to other animals. It also gives time for diseases to manifest before animals are assigned to a research project requiring long-term study.

Irrespective of their origin, animals will need some time to acclimatise to their novel home or experimental environment (review in: Grimpe 1928; Borrelli 2007; Borrelli and Fiorito 2008) before any experimental procedures can be contemplated, although the nature of the study may affect the duration of acclimatisation. Research is needed to identify objective measures of acclimatisation.

Acclimatisation also needs to be considered when moving animals from one tank to another within the laboratory especially if the animal has been moved out of water even for brief periods. For example, in *E. cirrhosa* Malham et al. (2002) showed that 5-min exposure to air produced a significant increase in plasma noradrenaline lasting up to 30 min and in reactive oxygen species lasting 2 h. The experimenter should be aware of potential handling and relocation stress, and their possible impacts upon their study. For instance, the skin of cuttlefishes and squids is delicate and may be harmed if they are removed from the water with nets; nautiluses are particularly sensitive to exposure to air (J. Basil, personal communication), and this should be avoided if possible by transporting them in vessels containing sea water; for octopuses, it is acceptable to use wet nets with a fine mesh (but see Walker et al. 1970). Movement of animals should be minimised.

### Environment and its control

#### Water supply and quality

As a minimum, sea water salinity, dissolved oxygen, pH, nitrogenous compounds and temperature must be monitored and maintained within physiological ranges reported for each species.

Cephalopod housing systems currently are predominantly based on open systems where a continuous supply of fresh sea water from a nearby location is available. More recently, efficient and relatively easily maintained closed aquarium systems have been developed (Toonen 2003; Gutnick et al. 2011b). In open sea water systems, water flow and exchange should be high enough to maintain water quality comparable to natural conditions. In a closed system, sea water salinity, dissolved oxygen, pH, nitrogenous compounds and temperature must be monitored and maintained within physiological ranges reported for each species.

Commercially available artificial seawater preparations are considered adequate and contain all the necessary substances and trace elements to keep cephalopods in good health (e.g. any mixture designed for marine invertebrates and corals but not fish is recommended). Trace elements, in particular strontium and calcium, should be monitored and added, if necessary.

Cephalopods are reported to accumulate (e.g.: Storelli et al. 2005; Seixas et al. 2005; Seixas and Pierce 2005; Raimundo et al. 2005; Napoleao et al. 2005; Raimundo and Vale 2008; Lacoue-Labarthe et al. 2008; Bustamante et al. 2008; Raimundo et al. 2009, 2010b; Pernice et al. 2009; Lourenco et al. 2009; Galitsopoulou et al. 2009; Pereira et al. 2009; Cirillo et al. 2010; Lacoue-Labarthe et al. 2012), and be sensitive to heavy metals (Raimundo et al. 2010a; de Polo and Scrimshaw 2012; Semedo et al. 2012), so care should be taken to ensure these are monitored and maintained within normal ambient ranges.

It is important to keep water and tanks clean of animal waste, uneaten food or inedible components (e.g. crab shells).

#### Light requirements

Photoperiod and light intensity should be maintained according to the natural living habits and possibly the geographical origin of the animal. A simulated dusk and dawn period is desirable. In the great majority of cases, cephalopods will adapt to changes in the lighting conditions in captivity (see for example: Fiorito et al. 1990; Borrelli 2007; Sykes et al. 2011). A number of studies have been carried out to analyse the circadian rhythm of several species (Houck 1982; Meisel et al. 2003, 2006; Brown et al. 2006; Frank et al. 2012). Recent studies also revealed an effect of light regimes on the growth of cuttlefish (Sykes et al. 2013). However, further studies are required to assess whether significant deviations in light intensity or photoperiod from the natural environment negatively impact animal welfare.

The use of a weak ambient light (e.g. moonlight lamp) or a specific red light illumination reduces the risk of disturbance when observation of the animal is required at night (e.g. Allen et al. 2010).

#### Noise and vibration

Recent evidence provides preliminary information on the impact of sound on cephalopods well-being (Guerra et al. 2007; André et al. 2011; Fewtrell and McCauley 2012). Noise, vibration and other sources of disturbance should be avoided; those originating from aquarium systems should be minimal, and preferably pumps and any other noise sources should be placed in a separate room.

### Assessment and maintenance of health and welfare

Animals must be inspected at least once a day by a competent person, and a record kept of their conditions (Directive Annex III requirement). Signs of health and illness in cephalopods vary with species (for a review of possible signs due to diseases see: Hochberg 1990; Hanlon and Forsythe 1990a, b). Signs based upon appearance, behaviour and physiology which could be used as part of health monitoring programme are summarised below. Criteria for identification of well-being and illness are closely related to the development of signs of pain, suffering, distress and lasting harm (PSDLH) required for assessment of the impact of regulated procedures and development of humane end points^5^ for studies (Andrews 2011a; Andrews et al. 2013) including assessment of the effect of surgical procedures or drug treatments on the animals. Signs of illness and PSDLH also need to be capable of some quantification to assess their magnitude and duration for implementing humane end points and reporting data (a requirement under the Directive, for publication by the EU) on the actual severity of effects caused by procedures (e.g. mild, moderate, severe) in comparison with that anticipated at the time of project evaluation. This is an area requiring considerable research, and the criteria outlined below should be viewed as a starting point, from which more detailed guidance is being developed (for details see Andrews et al. 2013).

For each of the categories below, consideration needs to be given to grading the signs to link to the assessment of severity. For example, what degree of weight loss would be considered mild, moderate and severe?

#### Indicators based on appearance of the animal

- Abnormal body colouration and body patterning, skin texture including swellings (bruising or oedema) and compromised skin integrity (erosion and ulceration); for examples see figures included in Hochberg (1990) and Hanlon and Forsythe (1990a, b). Skin lesions should always be closely monitored and if possible treated. For example, *E. cirrhosa* housed long term in the laboratory died within 2–4 days of the skin ulcerating (Boyle 1981, 1991).
- Abnormal morphology or damage to cuttlebone or shell.
- Abnormal body posture or position in the water column.
- Abnormal appearance as a result of body weight loss, possibly secondary to reduced food intake.

#### Indicators based upon the behaviour of the animal

- Reduced or absent food intake and a reluctance to feed or to attack (consider that reduced feeding during acclimatisation should be expected).
- Reduced or absent social interaction in social species and refusal or reluctance to leave a shelter in solitary housed species (e.g. *O. vulgaris*); lack of response to external stimuli or a sluggish response and in octopus a lack of natural curiosity.
- Stereotypic or repetitive behaviours.
- Reduced or excessive grooming and guarding behaviour of a body part.
- Abnormal motor or locomotor coordination.
- Autophagy or automutilation normally indicated by removal of one or more arms (Reimschuessel and Stoskopf 1990; Budelmann 1998).
- Excessive, uncontrolled or inappropriate inking.

#### Clinical signs

- Abnormal change (increase or decrease) in ventilation defined by rate, depth and coordination.
- Reduction in body weight over specific periods of time.
- Biomarkers such as phagocytes and catecholamines in the blood may increase due to several causes (e.g.: Malham et al. 1998a, b, 2002).^6^

### Causes of illness in cephalopods

Knowledge of the causes and diagnosis of illness (taken here to be any deviation from normal functionality) in cephalopods is rudimentary in comparison with fish and other vertebrates. The Directive’s requirement for health monitoring should act as a stimulus to research in this area and in particular systematically collection, collation and exchange data. It will be important to distinguish between illness acquired in the wild, acquired in the laboratory and congenital defects. Causes of illness can be summarised under the following headings, but each cause requires detailed research, as do treatments.

1. *Physical trauma* This includes skin, shell, pen or gladius and cuttlebone damage during capture of wild caught animals or by collision with a transport or holding tank wall (Grimpe 1928; Boyle 1981). Bites and limb amputation are also commonly observed in wild caught animals (e.g. *O. vulgaris*, Florini et al. 2011) but may also occur during fighting, cannibalistic behaviour (Ibáñez and Keyl 2010) or by autophagy/automutilation (Reimschuessel and Stoskopf 1990; Budelmann 1998).
2. *Parasites, bacteria and viruses* Host defence mechanisms in cephalopods have been reviewed by Ford (1992) and recently by Castellanos-Martinez and Gestal (2013). In the words of Boyle, “Cephalopods carry a wide variety of parasites and symbionts which include viruses, bacteria, fungi, sporozoans, ciliates, dicyemids (mesozoa), monogeneans, digeneans, cestodes, acanthocephalans, nematodes, polychaetes, hirudineans, branchiurians, copepods and isopods” (Boyle 1991, p. 133). However, there appear to be few data on the health impact (if any) of these various organisms, although it is likely that bacteria and viruses are causal agents of illness particularly in senescing animals with compromised defences (Anderson et al. 2002; Pascual et al. 2010). The cases provided below represent only few examples.
  Bacteria have been isolated from skin lesions in octopus and squid (e.g.: Hanlon et al. 1984; Pascual et al. 2006) and infection of *Octopus joubini* with *Vibrio alginolyticus* induced skin ulceration in 2 days (as reported by Boyle 1991) and in *E. cirrhosa* a related *Vibrio* sp. (obtained from the diet) impaired skin would healing (Polglase et al. 1983; Bullock et al. 1987).
  The gastrointestinal coccidian parasite *Aggregata octopiana* is found in *O. vulgaris* and produces malabsorption syndrome impacting growth (Castellanos-Martinez and Gestal 2011; but see also Castellanos-Martinez and Gestal 2013). A related organism (*A. andresei*) has been identified in the flying squid, *Martialia hyadesi* (Gestal et al. 2005). Larval nematodes including *Ascaris* and *Anisakis* (commonly found in fish) and larval and adult trematodes are reported in many cephalopod species (Hochberg 1990; Pascual and Hochberg 1996), but the health impact (if any) on the cephalopod is not known (for review see also Castellanos-Martinez and Gestal 2013).
  The bacterium *Vibrio fischeri* has been studied extensively as it is a symbiont of the bobtail squid, *E. scolopes* (McFall-Ngai 1994; Nyholm et al. 2009; McFall-Ngai et al. 2010; Rader and Nyholm 2012; Collins et al. 2012; Nyholm and Graf 2012) and should not be considered as a disturbance.
3. *Toxic substances* As reviewed by Smith (2008) and Smith et al. (2008) toxic agents may originate from food and seawater. Food, particularly crustacea and bivalves, are a potential source of a number of neurotoxins including paralytic, diarrhoeic and neurotoxic shellfish toxins (Watkins et al. 2008; for review see for example: Wang 2008; see also Paredes et al. 2011). Although the clinical effects of these toxins on humans are clear, the impact (if any) on cephalopods is not known, but—in view of the number of brain behavioural studies in which cephalopods are used—studies of the potential effect of the neurotoxic substances (including amnesia inducing toxins reported in cephalopods; e.g.: Costa et al. 2005; Costa and Pereira 2010; Lage et al. 2012; Braid et al. 2012) are needed to assess whether this could be a confounding factor in some research studies. Sea water may become toxic from excess levels of heavy metals and environmental pollutants. Little is known of the sensitivity of cephalopods to specific agents (e.g.: Raimundo et al. 2010a; Semedo et al. 2012). Measurements of antioxidant enzyme activity (catalase, superoxide dismutase, and glutathione *S*-transferases) in the digestive gland have been shown to be markers of oxidative stress induced by metal accumulation in *O. vulgaris* (Semedo et al. 2012). Recent studies have also shown that ingested nanoparticles induce immune responses in the octopus (Grimaldi et al. 2013). Sea water may also become toxic if oxygen, carbon dioxide, pH and nitrogenous waste products are outside normal limits (e.g.: Gutowska et al. 2010a, b; Hu et al. 2011) particularly if accompanied by elevated temperature.

### Age estimation and senescence

Age estimation in cephalopods is essentially based upon direct methods (Semmens et al. 2004) and analysis of increments in internal structures (e.g.: Choe 1963; Bettencourt et al. 1996; Perez et al. 1996; Le Goff et al. 1998; Jackson and Moltschaniwskyj 1999; Bettencourt and Guerra 2000; Arkhipkin 2005; Hall et al. 2007; Ikeda and Kobayashi 2010; Hermosilla et al. 2010; Canali et al. 2011a, b; Lei et al. 2012; Arkhipkin and Shcherbich 2012; Raya et al. 2013). Further research is recommended to estimate age in cephalopods in vivo.

With age, cephalopods undergo the natural process of senescence, a process where the body appears to “shut down” in females after brooding (review in Rocha et al. 2001) and the animal begins to die. The clinical signs of animals in senescence include reduced or absent drive to eat, cloudy eyes and changed behaviour (Chichery and Chichery 1992a, b; Dumont et al. 1994; for review see also Anderson et al. 2002). Good record keeping of age may help to differentiate between animals that are affected by diseases or simply show signs of senescence.

The predictable onset of senescence in some species of cephalopods post-reproduction (Rocha et al. 2001; but see also Anderson et al. 2002) and the modulation of the process by the secretions from the optic gland (Wodinsky 1977) may make cephalopods a model for investigating the impact of senescence on the brain (see also: Chichery and Chichery 1992a, b; Dumont et al. 1994) and provide insights in neuroprotective mechanisms. Such studies would need to be justified in the project evaluation process and in particular the potential welfare issues regarding the care of senescent animals carefully considered (see Smith et al. 2013 for discussion).

## Housing and care

### Tank specification and location

Tank requirements (for review see also: Grimpe 1928; Hanlon et al. 1983; Boletzky and Hanlon 1983; Borrelli 2007) vary tremendously between species as do stocking densities. In some benthic species, the available bottom surface area is an important requirement, whilst in others the volume of water is of more relevance. Shape and size of tanks should accommodate the natural behaviour of the animals. For example, Nautiloids need to be provided with vertical space, but benthic cephalopods need to be given large surface areas rather than deep tanks, and pelagic species need sufficient space to swim. Smooth, curved walls are recommended at least for cuttlefish and squid. Annex III of the Directive requires that “All animals shall be provided with space of sufficient complexity to allow expression of a wide range of normal behaviour. They shall be given a degree of control and choice over their environment to reduce stress-induced behaviour”. Animals should be provided with dens and shelters based upon their natural requirements. Use of gravel as a substrate for benthic species is highly recommended, but not mandatory. Environmental enrichment is already part of the best practice in cephalopod care for experimental purposes (e.g.: Fiorito et al. 1990; Mather and Anderson 1999; Dickel et al. 2000; Anderson and Wood 2001; Poirier et al. 2004; Borrelli 2007; Borrelli and Fiorito 2008; Boal 2011). It is interesting to note that in the classic work by Grimpe (1928) gravel, pebbles and stones are recommended to facilitate self-construction of a refuge by animals. In addition, other species, such as medium-sized sea stars, should be accommodated in the tanks to facilitate reduction in remains of food and faeces (Grimpe 1928). This would provide the enriched type of environment considered to be good welfare practice.

Cephalopods can be kept in shared water systems and rooms with different cephalopods species or other marine organisms. In principle, there is no need for separate rooms for experimental treatments and housing, but this will depend upon the type of study. For example, it is strongly recommended that a standardised dedicated room is used for behavioural experiments, and it is not good practice to perform surgical procedures and euthanasia in the same room where animals are housed. Moreover, animals subjected to surgical lesions should not be placed in a tank where there is a possibility that any chemical signal can be detected by un-operated animals.

Note that Directive Annex III, section A, includes general requirements pertaining to all species and also section B, for fish, where most principles might also apply to cephalopods.

### Animal stocking

Solitary animals (e.g. *O. vulgaris*) should be kept separately. Annex III of the Directive states that social animals must be socially housed in stable groups of compatible individuals (e.g. squids), but interactions should be monitored and animals separated if there is evidence of non-compatibility. Some animals such as *Nautilus* are primarily solitary in the wild, but may be housed together at low densities. The social structures of many species (e.g. *S. officinalis*) are not yet known, but captive bred European cuttlefish adults, as well as hatchling and young of all sources can be kept in groups (A. Sykes, pers. communication).

### Routine animal care and maintenance

Animal care includes routine maintenance, husbandry, and animal handling. Handling procedures should be standardised within the laboratory (and field) to minimise experimental variability produced by different handlers, also taking into account that some animals may learn to anticipate handling procedures (Boycott 1954). As with any live animal, handling and human interaction should be kept to the minimum needed to meet daily care and experimental requirements, standardised and performed by trained staff only, to minimise stress. Handling and all human interactions should be recorded, as the amount, frequency and nature of the interactions can influence husbandry and the outcome of experiments (for a general review see: Davis and Balfour 1992). For octopuses, the effects of rough handling on the skin may not be apparent until several days (Wells 1962), and as mentioned above, skin lesions may be fatal (as reviewed in Boyle 1991) so this could have major consequences if the animal had been assigned to a study requiring long-term survival. Even for commonly used laboratory mammals, the effects of different handling techniques are still being discovered; for example, Hurst and West (2010) compared commonly used techniques of handling laboratory mice and showed marked differences in biomarkers of anxiety. For cephalopods, optimal handling protocols need to be identified for each species to minimise adverse effects, which can be a confounding factor in experiments.

### Feeding

Feeding regimes should fit the lifestyle, natural diet, and developmental stages of the animals (see reviews in: Boletzky and Hanlon 1983; Borrelli 2007; Sykes et al. 2011, 2012). Cephalopods are carnivorous and, with the exception of the *Nautilus*, are predatory, and therefore, the use of live food can be essential, although may require justification (Smith et al. 2013). There are many examples of species and life stages where live prey is the only food accepted, and the benefits outweigh the risk of disease from the food. Efforts are underway to develop artificial diets. Daily feeding is common practice, and higher frequencies might be needed for young animals. Over feeding is preferred as long as excess food is removed in a time frame fitting the feeding habits of the species (Oestmann et al. 1997) and does not overwhelm the capacity of the filter system of the tank. Cuttlefish and squid are especially sensitive to lack of food; dead food can be used as alternative to live in some species (e.g.: Domingues et al. 2004; Ferreira et al. 2010).

Research is needed to identify optimal nutritional requirements that ensure health and welfare of each of the common laboratory species of cephalopod at key life stages. In addition, studies are needed to understand the physiological impact of a reduction in food intake because of illness, as a consequence of a surgical procedure or pharmacological intervention and as part of a training protocol for example when food may be used as a positive reinforcement. The impact of a particular experimental protocol upon food intake is likely to be a key question in harm-benefit evaluation of a project because of the high metabolic rate of cephalopods.

### Identification and marking techniques

Most studies identify animals using individual housing, but some studies are done with groups of animals. In general, marking soft parts of cephalopods may have a deleterious effect on health and welfare and should be avoided. When scientifically necessary, individual marking may be performed, under anaesthesia, using for example fluorescent elastomer tags (Zeeh and Wood 2009; e.g. Sepioteuthis sp.: Ikeda et al. 2009; e.g. Octopus sp.: Barry et al. 2011; Brewer and Norcross 2012) or integrated archival tags including implanted microchips (in *O. vulgaris*: Estefanell et al. 2011; in *S. officinalis*: Wearmouth et al. 2013). For *Nautilus*, individual shell marking is preferred and can be done without anaesthesia (J. Basil, pers. communication).

In non-shelled cephalopods, there have been some reports of the use of unique natural patterns of individual animals as a means of identification (Huffard et al. 2008). The application of noninvasive methods for identification of individuals is important in the interests of animal welfare.

## Procedures

A procedure within the Directive (Article 3, 1) is defined as “Any use, invasive or noninvasive, of an animal for experimental or other scientific purposes, with known or unknown outcome, or educational purposes, which may cause the animal a level of pain, suffering, distress or lasting harm equivalent to, or higher than, that caused by the introduction of a needle in accordance with good veterinary practice”. Objective criteria will need to identified by which it is possible to determine whether a particular procedure causes pain, suffering, distress or lasting harm equivalent or higher than that caused by the skilled introduction of a needle. In addition, the Directive also makes specific references to humane methods of killing (Article 6) and the use of anaesthesia and analgesia (Article 14). The potential impact upon many aspects of cephalopod research in general and the broad range of neuroscience research in particular is considerable. To illustrate this, examples of published studies are listed in Table 4, which are now likely to be regulated under the scope of the Directive if performed in the EU. In this section, we focus on some specific aspects to illustrate some challenges to neuroscience research presented by the above aspects of the Directive.

**Table 4:** Examples of published research on cephalopods which if carried out in the EU would now be likely to come within the scope of Directive 2010/63/EU

Research topic or technique	References
Implantation of electromyographic electrodes under anaesthesia in cuttlefish fin muscle and recording from unanaesthetised animals	Kier et al. (1989)
Removal of optic glands under anaesthesia followed by recovery (study effect on senescence)	Wodinsky (1977)
Sampling of haemolymph usually under anaesthesia	Malham et al. (1998a), Collins and Nyholm (2010), Grimaldi et al. (2013), Locatello et al. (2013)
Implantation of a catheter into the dorsal aorta for administration of drugs to the brain	Andrews et al. (1981)
Investigation of the efficacy of different anaesthetic techniques and mechanisms of anaesthesia	Andrews and Tansey (1981), Messenger et al. (1985), Seol et al. (2007), Sen and Tanrikul (2009), Pagano et al. (2011), Goncalves et al. (2012), Gleadall (2013)
Implantation of electrodes for recording or stimulation into the brain under anaesthesia followed by investigation of the effects in the conscious animal	Chichery and Chanelet (1976), Brown et al. (2006), Shomrat et al. (2008), Zullo et al. (2009), Mooney et al. (2010), Shomrat et al. (2011)
Removal of an arm or a tentacle with or without anaesthesia to investigate regeneration or the acute tissue and behavioural response to injury	Lange (1920), Crook et al. (2011), Fossati et al. (2013), Tressler et al. (2013)
Administration of substances into the circulation via the branchial hearts or intramuscular routes or directly into the brain	Agnisola et al. (1996), Fiorito et al. (1998), Agin et al. (2003), Graindorge et al. (2008)
Tracing nerve pathways using marker injection under anaesthesia followed by recovery to allow marker transport	Gaston and Tublitz (2004), Tublitz et al. (2006)
Implantation of electronic tags for tracking movement in the wild	Wearmouth et al. (2013)
Noninvasive measurement of brain size and arm morphology under anaesthesia with or without recovery	Grimaldi et al. (2007), Margheri et al. (2011b)
Killing animals (including hatchlings) to remove tissue (e.g. arm, brain), for study in vitro (e.g. brain slices), histological and molecular studies particularly if the study involves “nonstandard” methods	Kier et al. (1989), Westermann et al. (2002), Hochner et al. (2003), Kier and Stella (2007), Mackie (2008), Hague et al. (2013)
Brain or peripheral nervous system lesions under anaesthesia followed by recovery	Fiorito and Chichery (1995), Sumbre et al. (2001), Graindorge et al. (2006, 2008)
Use of aversive stimuli (e.g. electric shock, bitter taste) in training protocols	Robertson et al. (1994, 1995, 1996), Darmaillacq et al. (2004), Borrelli (2007)
Deprivation of food for 5 days, feeding with barium sulphate labelled shrimps, constraint of the animal and exposure to X-rays for imaging gut contents	Westermann et al. (2002)
Exposure of an animal to a potentially “stressful” environment/stimulus as an experimental procedure; examples include a large moving shape, a larger conspecific, a predator, air or sea water with temperature or oxygen partial pressure outside the normal aquarium range or manipulation of natural photoperiod/light intensity. Noninvasive immobilisation (confinement) may also constitute a stressful stimulus. The intensity, duration and exposure frequency are all factors which need to be considered	Malham et al. (2002), Cole and Adamo (2005), King and Adamo (2006), Adamo et al. (2006), Kuba et al. (2006), Canali et al. (2011a)
Production of hatchlings with deleterious phenotypes/genotypes by exposure of the eggs to a harmful environment or mutagen or genetic manipulation	Rosa et al. (2012)

Note that not all examples relate to invasive or surgical procedures (see also Ponte et al. 2013 for other resources). Papers have been selected to illustrate the diversity of studies likely to be regulated, and no comment is made about whether a particular study would now be permitted by a particular national competent authority

### Pain, suffering, distress and lasting harm (PSDLH)

One of several drivers for the inclusion of cephalopods in the Directive was a review of the evidence relating to their ability to perceive pain (EFSA Panel on Animal Health and Welfare 2005). The criteria used in the EFSA report have recently been reviewed in detail (Andrews et al. 2013) as has nociception in invertebrates (Crook and Walters 2011). At the time of the EFSA report (2005), evidence for the existence of nociceptors in cephalopods was largely circumstantial. Recently, afferents with the characteristics of nociceptors sensitive to mechanical stimulation have been described in a squid and evidence provided for long-term sensitisation (Crook et al. 2013). However, there are major gaps in our knowledge of the central processing of the information arising from the nociceptors in invertebrates in general and cephalopods specifically (Crook and Walters 2011; Andrews et al. 2013). The anatomy of the afferent projections from the arms and various lobes of the brain has been described for *O. vulgaris*, *S. officinalis* and *L. vulgaris* (Budelmann and Young 1985, 1987), but again neurophysiological studies are needed to understand the central processing of information from well-characterised nociceptors. Until such studies are performed, “pain perception” (i.e. what the animal might “feel” as a result of nociceptor activation) in cephalopods will remain a contentious issue. However, from an animal welfare perspective, researchers should be mindful of stimuli likely to activate nociceptors in their experimental protocols and either justify their use or take action to mitigate the impact. Neurophysiological studies in combination with behavioural studies will also be required to identify substances with analgesic effects that can be used postoperatively and to identify the mechanism(s) by which substances with presumed general anaesthetic actions in cephalopods act.

In addition to the physiology and pharmacology of pain perception in cephalopods, objective criteria for the identification and measurement of pain are required as part of welfare assessment and in particular to assess the impact of any experimental intervention. Although a great emphasis is rightly placed upon pain, equal consideration needs to be given to other ways in which an animal may suffer, be in distress or be caused lasting harm in an experimental setting and ways in which they can be identified and measured. Examples of “non-painful” types of suffering could include isolation in social species, housing in a tank of inappropriate size or with no refuge or being caused fear and anxiety (see Hawkins et al. 2011b for other examples). A preliminary approach to monitoring PSDLH in cephalopods has been recently described (see Table 1 in Andrews et al. 2013) based upon the types of criteria that have been developed over many years for mammals (e.g. Morton and Griffiths 1985).^7^

### General anaesthesia

General anaesthesia is required for performing surgical procedures followed by recovery (e.g. selective brain or nerve lesions, implantation of telemetry devices) for some types of in vivo physiological study (e.g. reflex control of the cardio-respiratory system, investigation of somato-sensory processing) and to permit handling for veterinary investigation and treatment. Over the last century, a diverse range of substances has been used to induce general anaesthesia in cephalopods (Pagano et al. 2011; Goncalves et al. 2012; Gleadall 2013; Andrews et al. 2013), but there have been relatively few studies utilising objective criteria to define the anaesthetic state or the mechanism and site of action and little consideration has been given to the procedures used from a welfare perspective (e.g. how aversive are the agents used?). Recently, isoflurane as been tested as an anaesthetic in *O. vulgaris* (Di Cosmo, pers. communication), but more investigation is required.

All current techniques use immersion in sea water containing the anaesthetic agent. Magnesium chloride and ethanol, used either separately or in combination, are the most commonly used agents. Following Andrews et al. (2013), criteria for assessment of general anaesthesia in cephalopods include: (1) depression of ventilation and in some cases cessation, probably accompanied by reduced cardiac activity; (2) decrease in chromatophore tone (indicative of reduced drive to or from the sub-oesophageal chromatophore lobes); (3) reduced arm activity, tone and sucker adhesion (particularly octopus); (4) loss of normal posture and righting reflex; (5) reduced or absent response to a noxious stimulus. The last needs to be used with some care as in *O. vulgaris* arms removed from the body withdraw in response to a noxious stimulus (Hague et al. 2013). Studies are urgently required to understand the way in which the putative anaesthetic agents act on the nervous system to produce the above effects and to render the animal into a presumed state of insensibility and unconsciousness. The site and mechanism of action of general anaesthetics has been studied extensively in mammals (e.g. Angel 1993), but there are few studies in cephalopods (e.g.: Andrews and Tansey 1981; Messenger et al. 1985), although with their high degree of encephalization combined with a brain organised in a fundamentally different way from vertebrates studies of general anaesthesia may provide novel insights into mechanisms of consciousness.

### Humane methods of killing

The Directive requires that if it is necessary to kill an animal (e.g. at the end of project, to obtain tissue for an in vitro study, because a humane end point is reached), it must be done “with the minimum of pain, suffering and distress” (Article 6). Acceptable methods should comply with the general principles of humane animal euthanasia set out in Demers et al. (2006) and Annex IV of the Directive.

Identification of humane methods for killing is a particular challenge for neuroscience as physical destruction of the brain is a commonly used method and maybe acceptable if the method used can be demonstrated to be humane, but it is obviously not suitable when the brain is the subject of study. Similarly, overdose of general anaesthetic is often used, but again could be argued to compromise subsequent studies of brain function because of the residual pharmacological effect of the agent used and the effects of asphyxia caused by the prolonged (usually >30 min) immersion in anaesthetic needed to kill the animal. Such constraints may encourage investigation of electrical euthanasia methods similar to those used for crustaceans (Neill 2010). Annex IV of the Directive also includes methods for confirmation of death, and these are discussed in relation to cephalopods in Andrews et al. (2013). It should also be noted that the requirement for humane killing also applies to hatchlings. In this last case, killing by direct immersion in fixative would not now be considered acceptable in the EU, although it might be possible to obtain permission to use this as a method if it could be justified to the NCA.

Humane killing methods for both hatchlings and developmental stages through adult cephalopods require additional research, but in the *interim* it is proposed that animals are either anaesthetised prior to mechanical destruction of the brain (this may be difficult in nautiloids) or if the brain is required that animals (including hatchlings) are killed by prolonged immersion in anaesthetic, recognising that the impact upon the brain will need to be considered in the light of the scientific objectives and that a shorter period of anaesthesia followed by decapitation when the animal is insensible may need to be considered (Andrews et al. 2013).

## Replacement, refinement and reduction and cephalopod research

The principles of Replacement, Refinement and Reduction (“3Rs”) developed by Russell and Burch (1959) as key elements of humane experimentation involving sentient animals are at the heart of the Directive (Article 4), and project evaluation prior to authorisation requires an assessment of how the 3Rs are addressed in the proposed study (Article 38). Replacement “of the use of a regulated living animal” is often used in the context of pharmaceutical research to describe replacing a test (e.g. for drug efficacy) in a living animal with one using a microorganism, human tissue or in silico methods. Superficially, “replacement” may not appear to apply to most cephalopod research as many researchers are undertaking the research because they have a specific interest in an aspect to cephalopod biology, but “replacement” requires researchers to consider a priori whether they need to use a “living animal” (of a species covered by the Directive) to answer the specific research question or whether the same research question could be tackled in another way which could e.g. include in vitro studies of tissue take from the same species provided that the animal is killed using an approved humane method.

Most researchers will already be applying the principle of “reduction” to their studies as this relates to the use of the minimum number of animals required to achieve the scientific objectives of the project and is an inherent part of good experimental and statistical design. Although not strictly an example of “reduction” within the meaning of the 3Rs the Directive also makes a specific point (Article 18) about sharing organs and tissues from killed animals. This could contribute to a reduction in the overall number of animals used within an institute by coordinating the killing animals at the end of a procedure with in vitro studies requiring fresh living tissue and/or banking tissue for molecular or histological studies. In the case of the latter, tissue could be shipped to other institutions.

Refinement is the “R” most likely to impinge upon current cephalopod research by requiring that experimental procedures, housing, husbandry and all aspects of care are “refined” so that they cause the minimum possible pain, suffering, distress or lasting harm throughout the life of the animal being used. Refinement of current best practice in the care and welfare assessment of the various cephalopod species will evolve by research to provide evidence to support changes of approach and technique that reduce adverse effects and maybe informed by approaches to refining procedures commonly carried out on laboratory vertebrates (Hawkins et al. 2011a). For experimental procedures, refinement requires the researcher to carefully examine their protocols and see where changes in can be made in any aspect likely to cause PSDLH to reduce adverse effects whilst achieving the scientific outcome. For example, it might be asked whether the number of haemolymph samples taken or number of drug doses given each day could be reduced; or whether positive reinforcement could be used instead of negative ones in training protocols; and whether induction of general anaesthesia could be made more humane by exposing the animal to a gradually rising concentration of anaesthetic rather than direct immersion in a fully effective concentration. All three are examples of approaches accepted and used to refine procedures in vertebrates. Additional examples using hypothetical research projects involving cephalopods are discussed in Smith et al. (2013).

## Conclusion

Directive 2010/63/EU is a milestone for invertebrate research in the EU because it is the first time particular types of research involving an entire class of invertebrates, the cephalopods, will be regulated in the same way as scientific projects involving vertebrates. Although regulation presents challenges, there are several areas where neurophysiological and behavioural neuroscience research could be useful to address key questions related to cephalopod care and welfare discussed above. Most researchers already recognise the relationship between good welfare and good science, but the development of consensus Guidelines for Care and Welfare of Cephalopods led by the research community will facilitate the dissemination and adoption of good practice. Guidelines are being developed based upon literature review and discussion meetings, but they are only an initial step and evolution of such guidelines will rely upon capturing the experience and knowledge of the cephalopod research community. It is hoped that this review will prompt readers to investigate some of the neuroscience questions posed and to contribute to the future development of guidelines for optimal care and welfare of cephalopods via publication and contributions to online research fora (e.g. CephRes: www.cephalopodresearch.org; CephSeq: http://cephseq.org/; Cephalopod International Advisory Council: http://www.abdn.ac.uk/CIAC/).

## Footnotes

1 The vertebrate species commonly covered were those utilised in experiments (primarily physiology) at the time (e.g. frogs, cats, dogs). The wording of the UK 1876 Cruelty to Animals Act perhaps gives an additional insight into the basis for “the division” between invertebrates and vertebrates. The Act permits “the advancement of new discovery of physiological knowledge by experiments calculated to *give pain*” (our italics), implying that the authors of the Act may have taken the view that whilst vertebrates may experience pain invertebrates do not (but see Fiorito 1986; and Andrews 2011a; Andrews et al. 2013 for details).

2 A list of NCA is available at http://ec.europa.eu/environment/chemicals/lab_animals/ms_en.htm.

3 For an updated list see Journals that have incorporated ARRIVE in their Instructions for Authors at: http://www.nc3rs.org.uk/page.asp?id=1796.

4 Developing guidelines for the care and welfare of Cephalopods under European directive 2010/63/EU. Available at: http://www.felasa.eu/announcements/felasa-collaboration-on-cephalopods.

5 I.e. the predetermined criteria which if they are reached result in termination of the procedure or require treatment/euthanasia of the animal.

6 Note there is also a possibility of measuring faecal steroids (e.g. cortisol) as well as reproductive hormones (Larson and Anderson 2010) to assess welfare with the advantage that such techniques are noninvasive.

7 For severity assessment see also: http://ec.europa.eu/environment/chemicals/lab_animals/pdf/Consensus%20doc%20on%20severity%20assessment.pdf.
